# A Scoping Review: Ketamine for the Prevention of Perioperative Shivering in Patients Undergoing Spinal Anesthesia

**DOI:** 10.7759/cureus.66630

**Published:** 2024-08-11

**Authors:** Kenneth Goich, Dakota Pastore, Bianna Koutsenko, Benjamin Infosino, Mitchell N Sgrignoli, Todd Schachter

**Affiliations:** 1 Medicine, Rowan-Virtua School of Osteopathic Medicine, Stratford, USA; 2 Family Medicine, Rowan-Virtua School of Osteopathic Medicine, Stratford, USA

**Keywords:** perioperative hypothermia, intraoperative shivering, perioperative shivering, post spinal anaesthesia shivering, shivering, ketamine

## Abstract

Shivering is a frequently encountered perioperative complication in patients undergoing spinal anesthesia. Numerous different pharmacological agents have been employed to mitigate this issue. This scoping review aims to evaluate the efficacy of ketamine in mitigating the incidence of shivering. This review process utilized PubMed, JAMA, and Cochrane as primary databases. Searches were performed using combinations of key terms: "Ketamine," "Shivering," "Spinal Anesthesia," and "Hypothermia." Reviews of reference lists for additional pertinent data were performed. When ketamine was compared against a saline control, three out of five studies found ketamine to be more effective (p < 0.05, p < 0.001, p < 0.001) in the prevention of shivering. When compared with tramadol, two studies found ketamine to be more effective (p < 0.001, p < 0.001), one found no difference (p = 0.261), and one found tramadol to be more effective (p < 0.001). Two studies found dexmedetomidine more effective (p < 0.022, p < 0.027) than ketamine and tramadol. When comparing ketamine, ondansetron, and meperidine, all three were effective (p < 0.001) versus saline, with no significant difference between the three. Meperidine demonstrated more efficacy (p < 0.05) in reducing the intensity of shivering than ketamine. Ketamine's effects on hemodynamics were shown to be equivocal or more favorable across several studies. While there is mixed evidence on whether it is better than other treatments, ketamine may have advantages from a hemodynamic standpoint. Dosages of 0.2-0.5 mg/kg with or without a subsequent infusion of 0.1 mg/kg per hour may aid in the prevention of perioperative shivering. Overall, ketamine is a safe and effective drug for the prevention of perioperative shivering. However, other drugs may be equally or more effective; therefore, patient population, hemodynamic status, patient preferences, and provider familiarity with different agents should be considered.

## Introduction and background

Shivering is an involuntary somatic muscle response typically triggered by prolonged exposure to cold environments or fever to raise body temperature by generating heat through repetitive contraction of skeletal muscles. Shivering is primarily controlled by the median preoptic nucleus (MnPO) in the anterior thalamus of the brain which contains the central efferent pathways for cold-defensive and febrile shivering. Some common causes of shivering include movement disorders, excitement, fear, stress, tremors, low blood sugar, anxiety, fever, cold exposure, postanesthetic shivering, and shivering with spinal anesthesia.

Patients frequently experience shivering following surgery with general or spinal anesthesia. This shivering may be due to a natural thermoregulatory response to central hypothermia or as a result of the release of cytokines throughout the surgical process [1]. This is unpleasant for the patient and occurs following surgery in 30-65% of patients who have received general anesthetics [1].

The exact mechanism underlying post-spinal anesthesia shivering is not fully understood but may involve thermoregulatory responses to hypothermia, affecting neurons in specific brain regions. Shivering with spinal anesthesia is an involuntary, oscillatory muscular activity that significantly increases metabolic heat production by up to 600% and increases oxygen consumption up to 400% [2]. This may lead to arterial hypoxia and is associated with an increased risk to patients with myocardial infarction [1]. These sequelae of shivering may prolong post-operative recovery time and contribute to poor patient outcomes.

 A variety of medications have been studied to prevent or treat post-anesthesia shivering; recent studies indicate ketamine shows promise in controlling shivering. Ketamine is a competitive N-methyl-D-aspartate (NMDA) receptor antagonist and is involved in the regulation of heat. As a NMDA agonist, it increases the rate of neuronal discharge in the anterior hypothalamic preoptic region modulating serotonergic and noradrenergic neurons in the locus coeruleus [3]. The mechanism of action by which ketamine controls shivering has yet to be determined, but it is believed that it regulates shivering by producing non-vibration-induced heat, acting on the hypothalamus and beta-adrenergic effects. 

As there is not yet a determined most effective agent this scoping review of current literature was conducted to determine the benefits of ketamine in the prevention of perioperative spinal anesthetic shivering. Hemodynamic effects of ketamine and other anesthetic agents were examined as a secondary objective.

## Review

Methods 

A scoping review of the current literature was conducted to determine the benefits of ketamine in the prevention of perioperative spinal anesthetic shivering. The hemodynamic effects of ketamine and other anesthetic agents were examined as a secondary objective.

Information Sources and Search Terms Strategy

The medical databases used for ascertaining data included PubMed and Cochrane. Using search terms “ketamine” AND “shivering” AND “spinal,” “ketamine shivering spinal,” “ketamine” AND “hypothermia” AND “spinal,” and “ketamine shivering,” a total of 104 articles were collected, 52 from PubMed, four from JAMA, and 48 from Cochrane (Table 1). This search was performed by authors (KG and DP) between December 27, 2023, and March 5, 2024. Reviews of reference lists for additional pertinent data were performed.

**Table 1:** Search results

Database	Search Terms	Number of articles
PubMed	“Ketamine” AND “shivering” AND “spinal”	13
	“ketamine shivering spinal”	13
	“ketamine” AND “hypothermia” AND “spinal”	3
	“ketamine shivering”	23
JAMA	“Ketamine” AND “shivering” AND “spinal”	2
	“ketamine shivering spinal”	2
Cochrane	“Ketamine” AND “shivering” AND “Spinal”	48

Data Extraction

Studies published during the years 2018 to 2024 were used for data gathering. Results, conclusions, and methodology were extracted from the collected literature. Of the total 104 articles discovered during the data search, 13 studies met the inclusion criteria.

Inclusion Criteria

Only papers written in the English language were included in the scoping review. The review included observational, descriptive, and experimental studies. The types of studies included in the analysis were randomized controlled trials and cohort studies. Articles were included regardless of nation of origin.

Exclusion Criteria

Articles excluded from the study were those with full text unavailable, not in the English language, not published in a peer-reviewed journal, studies still in progress without reported data, and those published prior to 2018. Articles not including the terms "ketamine" and "shivering" or "hypothermia" and "spinal" were excluded.

Data Analysis

No statistical methods were used to interpret the data. All studies meeting inclusion criteria were reviewed independently by authors (KG and DP). Findings and analysis of collected literature were provided in the results, discussion, and conclusion sections of the proposed literature review. 

Results

Ketamine Versus Saline

When compared against saline alone, ketamine has been shown to be effective in the prevention of shivering in operative patients undergoing spinal anesthesia. In patients undergoing spinal anesthesia for cesarean delivery (CD), ketamine has shown mixed results in its efficacy as a method of preventing shivering.

In a double‐blind, randomized placebo-controlled trial, Sarshivi et al. [2] examined the efficacy of 0.3 mg/kg of IV ketamine versus a saline control group in the prevention of shivering of women immediately after CD (Table 2). Shivering was graded on a 0-3 scale, a score of 2 (muscle activity in > 1 group but not generalized) or 3 (generalized muscle activity) being considered intervention failure, warranting 30 mg of IV meperidine to terminate shivering [2]. In the ketamine group, 33.3% (15/45) of patients displayed shivering scaled 2 or more versus 53.3% (24/45) in the saline control group [2]. While the ketamine group displayed fewer incidences, this value was not statistically significant (p = 0.08, 95% CI: 0.62 (0.38-1.02) [2]. In a comparison of the intensity of shivering (median quartiles 1 and 3), the ketamine group, 0(0-2), versus the saline group, 1 (0-2), showed ketamine to significantly reduce shivering intensity (p = 0.034) [2].

**Table 2:** Ketamine versus saline

First Author	Administration	N, Total	N, Saline	N, Ketamine	Dose Ketamine	Shivering, Saline	Shivering Ketamine	P value	Notes
Sarshivi [2]	IV	90	45	45	0.3 mg/kg	24 (53.3%)	15 (33.3%)	0.08	
Xue [3]	Epidural	60	30	30	0.5 mg/kg	10 (33.3%)	2 (6.67%)	< 0.05	At 30 minute mark
Aboelsed [4]	IV	126	63	63	0.3 mg/kg bolus	22 (38.1%)	5 (7.94%)	< 0.01	
					0.1 mg/kg per hour infusion				
Adhikari [5]	IV	80	40	40	0.25 mg/kg	8 (20%)	5 (12 %)	0.36	
Thangavelu [6]	IV	60	31	29	0.2 mg/kg	18 (58.06%)	4 (13.79%)	< 0.01	

Using a scale grading shivering from 0 to 4 based on severity, with rescue with 25 mg IV meperidine being given when shivering > 3 (muscle activity in > 1 muscle groups), Xue et al. [3] found ketamine to have a statistically significant (p < 0.05) effect on decreasing the incidence and intensity of shivering, specifically at the 30-minute mark (Table 2). In their prospective, randomized, double-blind study, a single dose of 0.5 mg/kg of epidural ketamine was administered to 30 patients who underwent spinal anesthesia for CD to prevent intraoperative shivering with comparative measurements against a control of saline occurring at different time periods [3]. Ketamine and saline were comparable except at the 30-minute mark, where the rate of shivering in the ketamine group was 6.67% versus the saline group with a shivering rate of 33.3% [3].

In a randomized, double-blind, prospective, controlled study, Aboelsuod et al. [4] found that an IV ketamine dose of 0.3 mg/kg bolus, followed by a 0.1 mg/kg per hour infusion, was effective for the prevention of shivering in women undergoing CD with spinal anesthesia (Table 2). The ketamine group was compared against a control group that received saline, primarily to examine the effects of ketamine on hemodynamics [4]. In addition to the evaluation of the hemodynamic effects of ketamine, the incidence of shivering was reported to be 7.94% (5/63) in the ketamine group versus 38.1% (22/63) in the saline control group, showing a statistically significant difference (p < 0.01) [4].

Adhikari et al. [5] performed a prospective, randomized, double-blind study primarily evaluating the analgesic effects of IV ketamine versus saline in patients post CD who underwent spinal anesthesia, by measuring the amount of morphine needed for pain control (Table 2). Along with analgesia, the incidence of shivering was reported upon utilizing the Tsai Chu shivering scale, and 20 mg of meperidine was administered for shivering involving the entire body [5]. Their study showed that in the ketamine group, 12% (5/40) of patients required meperidine for shivering, versus 20% (8/40) requiring intervention in the control group; however, this difference was not statistically significant (p = 0.36) [5].

In a randomized, double-blind control trial, Thangavelu et al. [6] studied the incidence of shivering following prophylactic IV ketamine versus saline in 60 patients undergoing abdominal or lower limb surgery (Table 2). A ketamine dose of 0.2 mg/kg IV bolus, followed by 0.1 mg/kg per hour, was compared to a saline bolus, followed by an infusion that functioned as a control [6]. In patients who underwent shivering, intensity defined as “muscular activity in more than one group but not generalized” or “shivering involving the whole body,” the prophylaxis was considered a failure, and 0.1 mg/kg IV Tramadol was given as a rescue drug [6]. The incidence of intraoperative shivering requiring rescue in the 31-patient control group was 58.06% (18) versus 13.19% (4) in the ketamine group (p < 0.01) [6]. Patients were monitored for two hours postoperatively, and among those that did not require rescue due to shivering, all patients in the ketamine group continued to not shiver, versus 30.77% of patients in the saline group who developed significant shivering during the postoperative period (p < 0.01) [6].

Ketamine Versus Tramadol

A randomized controlled trial studying IV ketamine versus IV tramadol in the prevention of intraoperative shivering in patients who underwent CD under spinal anesthesia was performed by Azam et al. [7] (Table 3). In the 200-patient ketamine group, a prophylactic 0.5 mg/kg IV dose of ketamine was given versus a 2 mg/kg IV dose of tramadol in another 200-patient group [7]. In the ketamine group, shivering occurred in 39 (19.5%) versus 72 (36%) in the tramadol group, demonstrating a statistically significant (p < 0.001) decrease in the incidence of shivering with administration of ketamine compared to tramadol [7].

**Table 3:** Ketamine versus tramadol * values in table at 60 minute mark

First Author	Administration	N, Total	N, Tramadol	N, Ketamine	Dose Tramadol	Dose Ketamine	Shivering, Tramadol	Shivering Ketamine	P value	Notes
Azam [7]	IV	400	200	200	2 mg/kg	0.5 mg/kg	72 (36%)	39 (19.5%)	<0.001	
Gemechu [8]	IV	516	258	258	0.5 mg/kg	0.25 mg/kg	113 (43.8%)	74 (28.7%)	0.001	
Ahmed [9]	IV	200	100	100	1 mg/kg	0.5 mg/kg	6 (6%)	32 (32%)	<0.001	*at 60 minute mark. 15 min: Tramadol - 8% (8); Ketamine 42% (42); p<0.001 30 min: Tramadol - 7% (7); Ketamine 35% (35); p<0.001 45 min: Tramadol - 7% (7); Ketamine 36% (36); p<0.001
Seyam [10]	IV	150	50	50	0.5 mg/kg	0.2 mg/kg	28 (56%)	18 (36%)	0.261	Other N=50 is the saline group, both Tramadol and Ketamine showed a statistically significant (p=0.03) decrease in shivering versus the Saline group

Gemechu et al. [8] performed a prospective cohort study comparing prophylactic IV 0.25 mg/kg ketamine versus IV 0.5 mg/kg tramadol to prevent intraoperative shivering in patients who underwent orthopedic surgery under spinal anesthesia (Table 3). In the ketamine group, the shivering occurred in 28.7% (74) of patients versus 43.8% (113) of patients in the tramadol group, showing ketamine provided a statistically significant (p = 0.001) better outcome [8].

In a prospective, randomized, double-blind, placebo-controlled study of 150 patients, Seyam [9] examined the efficacy of ketamine versus tramadol for the prevention of intraoperative and postoperative shivering for patients who received spinal anesthesia for elective surgeries (Table 3). Subjects were divided into three groups of 50 patients each, and after spinal anesthesia was given, patients received IV 0.2 mg/kg ketamine or IV 0.5 mg/kg tramadol or IV 5 mL saline [9]. Shivering was reported on a 5-point scale every five minutes intraoperatively and 10 minutes post-operatively for 60 minutes [9]. Patients who underwent shivering within 15 minutes of receiving spinal anesthesia graded as 3 (more than one muscle group but not generalized) or 4 (shivering involving the whole body) were given IV 25 mg meperidine for rescue [9]. The incidence of shivering was statistically significantly less (p = 0.03) in the ketamine (7 (14%)) and tramadol (11 (22%)) groups versus the saline group (22 (44%)) [9]. There was no statistical significance (p = 0.261) between ketamine and tramadol groups when compared to each other [9].

Ahmed et al. [10] compared the efficacy of IV ketamine versus IV tramadol for the prevention of shivering in patients who underwent abdominal surgeries under spinal anesthesia (Table 3). A 100-patient ketamine group received a prophylactic IV 0.5 mg/kg dose for comparison against 100 patients who received prophylactic IV 1 mg/kg of tramadol [10]. Shivering between groups was compared at 15-minute time intervals for one hour, with 60 minutes being the primary focus [10]. Tramadol had a statistically significant (p < 0.001) greater efficacy at all time intervals, with the 60-minute mark showing shivering in 6% (6) of patients versus 32% (32) of patients in the ketamine group [10].

Ketamine Versus Tramadol Versus Dexmedetomidine

In a randomized controlled trial of 200 patients who received spinal anesthesia, Ameta et al. [11] divided patients into four groups, IV 0.5 mg/kg tramadol, 0.5 mg/kg ketamine, 0.5 mcg/kg dexmedetomidine, or 10 mL saline, to compare their efficacy in the prevention of shivering (Table 4). The incidence of shivering was evaluated on a 0-4 scale, with 0 being no shivering and 4 being whole body shivering with shivering grades of 3 and 4 receiving IV 25 mg meperidine for rescue [11]. Group differences showed the incidence of shivering in the dexmedetomidine group (12 (24%)) to be statistically significantly less (p = 0.022) when compared to ketamine (23 (46%)), tramadol (25 (50%)), and saline (21 (42%)) [11]. The necessity for rescue meperidine was 6% in the dexmedetomidine group versus 12% in the ketamine group, 20% in the tramadol group, and 16% in the saline group [11].

**Table 4:** Ketamine versus tramadol versus dexmedetomidine

First Author	Administration	N, Total	N, Tramadol	N, Ketamine	N, Dexmedetomidine	N, Saline	Dose Tramadol	Dose Ketamine	Dose Dexmedetomidine	Dose Saline	Shivering, Tramadol	Shivering, Ketamine	Shivering, Dexmedetomidine	Shivering Saline	P Value
Ameta [11]	IV	200	50	50	50	50	0.5 mg/kg	0.5 mg/kg	0.5 mcg/kg	10 mL	25 (50%)	23 (46%)	12 (24%)	21 (42%)	0.022
Khan [12]	IV	120	30	30	30	30	0.5 mg/kg	0.25 mg/kg	0.5 mcg/kg	5 mL	10 (33.3%)	2 (6.6%)	0	11 (36.6%)	0.027

In a randomized clinical trial of the efficacy of IV ketamine versus IV tramadol versus IV dexmedetomidine versus saline in the prevention of shivering, Khan et al. [12] studied 120 patients who underwent spinal anesthesia for surgery (Table 4). Patients were divided into four groups of 30, receiving 0.5 mg/kg tramadol, 0.25 mg/kg ketamine, 0.5 mcg/kg dexmedetomidine, or 5 mL saline [12]. In the dexmedetomidine group, no patients exhibited shivering, a statistically significant (p = 0.027) decrease when compared to tramadol (10 (33%)), ketamine (2 (6.6%)), and saline (11 (36.6%)) [12].

Ketamine Versus Meperidine

In a double-blind randomized clinical trial, Gholinataj et al. [13] compared the efficacy of IV ketamine versus intrathecal meperidine in the prevention of post-anesthetic shivering following spinal anesthesia in patients who underwent lower limb orthopedic surgeries. The trial included 150 patients divided into three groups of 50. In one group, patients received IV 0.5 mg/kg ketamine, in another group, patients received intrathecal 0.2 mg/kg meperidine, and in the control group IV 5mL saline was administered [13]. The intensity of shivering was graded using a scale from 0 to 4 and evaluated three minutes before the onset of spinal anesthesia, as well as 20, 60, 80, 100, and 120 minutes after the onset of spinal anesthesia [13]. In the initial comparison at three minutes, there was no significant difference between groups; however, at the 20-minute mark, and in all subsequent time comparisons, the saline control group showed a statistically significant greater intensity of shivering, with meperidine being the most effective (p < 0.05) [13].

Ketamine Versus Other Agents

Ramanathan et al. [14] performed a randomized controlled study comparing the efficacy of ketamine versus ondansetron versus meperidine versus saline for the prevention of shivering in 203 patients who received spinal anesthesia for elective total knee replacement surgery (Table 5). There were 51 patients in the ketamine group who received an IV dose of 0.25 mg/kg, 50 patients in the ondansetron group who received an IV 4 mg dose, 52 patients in the meperidine group who received an IV 0.25 mg/kg dose, and 50 patients in the control group that received 10 mL of IV saline [14]. They recorded the incidence and severity of shivering during the procedure at five-minute intervals and graded the severity on a scale of 0-4 [14]. A grade of 2 (muscular activity in one muscle group) or more at least 15 minutes after spinal anesthesia was considered shivering, and a grade of 3 (muscular activity in more than one group but no generalized) or 4 (shivering involving the whole body) at least 15 minutes after spinal anesthesia was considered prophylaxis failure, resulting in the administration of IV 50 mg tramadol for rescue [14]. Overall comparison of the incidence of shivering between ketamine (4 [7.84%]) and ondansetron (8 (16%)) and meperidine (4 (7.69%)) and saline (22 (44%)) was statistically significant (p = 0.001) with each individual agent showing a statistically significant efficacy versus the saline control in the individual comparisons (ketamine vs saline, p = 0.001; ondansetron vs saline, p = 0.002; meperidine vs saline, p = 0.001) [14]. While agents showed significant efficacy versus control, intergroup comparisons were not statistically significant (ketamine vs ondansetron, p = 0.15; ketamine vs meperidine, p = 0.88; ondansetron vs meperidine, p = 0.14) [14].

**Table 5:** Ketamine versus ondansetron versus meperidine versus saline Data from Ramanathan et al. [14].

Drug	Administration	Dose	N	N, Shivering	P	P vs Ketamine	P vs Ondansetron	P vs Meperidine	P vs Saline
Ketamine	IV	0.25 mg/kg	51	4 (7.84%)	0.001		0.15	0.88	0.001
Ondansetron	IV	4 mg	50	8 (16%)		0.15		0.14	0.002
Meperidine	IV	0.25 mg/kg	52	4 (7.69%)		0.88	0.14		0.001
Saline	IV	10 mL	50	22 (44%)		0.001	0.002	0.001	

Hemodynamics

Sarshivi et al. [2], Xue et al. [3], and Adhikari et al. [5] reported the incidence of hypotension in ketamine versus saline groups (Table 6). When incidence was compared in these studies, only Xue et al. [2] showed a statistically significant difference between groups (p < 0.05) with 10% (3/30) in the ketamine group becoming hypotensive versus 30% (10/30) in the saline control group. Sarshivi et al. showed a 60% (27/45) incidence of hypotension in the saline group versus 55.6% (25/45) in the ketamine group and Adhikari et al. [5] with 40% (16/40) incidence in the saline group versus 32% (13/40) in the ketamine group; however, these differences were not statistically significant (p = 0.67 and p = 0.48, respectively). Additionally, Sarshivi et al. [2] reported the incidence of bradycardia as 6.7% (3/45) in the saline group and 0 in the ketamine group; however, these differences were not significant either (p = 0.24) (Table 7).

**Table 6:** Incidence of hypotension

First Author	Saline Control	Ketamine	Ondansetron	Meperidine	Tramadol	P value
Sarshivi [2]	27/45 (60%)	25/45 (55.6%)				0.67
Xue [3]	10/20 (33%)	3/30 (10%)				< 0.05
Adhikari [5]	16/40 (40%)	13/40 (32%)				0.48
					Ramanathan [14]		24/50 (48%)	8/51 (15.7%)	24/50 (48%)	27/52 (51.9%)		< 0.001
Seyam [9]	9/50 (18%)	9/50 (18%)			11/50 (22%)	0.842

**Table 7:** Incidence of bradycardia

First Author	Saline Control	Ketamine	Ondansetron	Meperidine	Tramadol	P value
Sarshivi [2]	3/45 (6.7%)	0/45				0.24
Ramanathan [14]	6/50 (12%)	3/51 (5.6%)	4/50 (8%)	5/52 (9.6%)		0.74
Seyam [9]	0/50	0/50			0/50	

In Ramanathan et al.'s [14] comparison between saline, ketamine, ondansetron, and meperidine for the prevention of shivering, they also reported on the incidence of hypotension and bradycardia (Table 6 and Table 7). The incidence of hypotension in the saline group was 48% (24/50), 15.7% (8/51) in the ketamine group, 48% (24/50) in the ondansetron, and 51.9% (27/52) in the meperidine group, demonstrating a significant (p = 0.001) decreased incidence of hypotension in the ketamine group [14]. There was however no significant (p = 0.74) difference in the incidence of bradycardia [14].

Seyam [9] compared the incidence of hypotension and bradycardia in ketamine, tramadol, and saline (Table 6 and Table 7). The incidence of hypotension in the ketamine (9/50 (18%)) versus tramadol (11/50 (22%)) versus saline (9/50 (18%)) was shown to have no statistically significant difference (p = 0.842) [9]. There were no reported incidences of bradycardia among any group [9].

Aboelsuod et al. [4] monitored for bradycardia and hypotension at 10-minute intervals in their comparison between saline and ketamine, with ketamine showing a significantly decreased incidence of hypotension at the 10-minute, 20-minute, and 30-minute intervals (p = 0.02, p = 0.004, p = 0.002, respectively) (Table 6 and Table 7). Other time frames saw no significant difference. Ketamine also showed a significant decrease in the incidence of bradycardia at the 10-minute, 20-minute, and 30-minute intervals (p = 0.04, p = 0.02, p = 0.04, respectively), with no significant difference at further measurements [4]. 

Gholintaj et al. [13] reported on their monitoring of hypotension and bradycardia at 20-minute intervals for two hours in their comparison between ketamine, meperidine, and saline (Table 6 and Table 7). Ketamine showed a significantly higher average mean arterial pressure (MAP) at the 20-minute (MAP = 100.32) and 60-minute (MAP = 96.26) intervals (p = 0.019 and p = 0.047, respectively), as well as a significantly higher mean heart rate at the 20-minute (HR = 90.75) and 60-minute (HR = 85.64) intervals (p = 0.013 and p = 0.027, respectively). Other intervals demonstrated no significant difference in MAP or heart rate [13].

When comparing ketamine and tramadol, Gemechu et al. [8] reported at 10-minute intervals for one hour and demonstrated a significantly (p = 0.001) higher mean MAP (83.75, 83.41, 83.34, 83.64, 83.91, respectively) from the 20-60 minute mark and significantly (p = 0.001, p = 0.001, and p = 0.007, respectively) higher mean HR (80.22, 80.42, 81.09, respectively) at the 20-minute, 30-minute, and 40-minute measurements [8].

Discussion 

Perioperative shivering is a possible complication in patients receiving spinal anesthesia. Along with other complications such as hypothermia and hypotension, shivering is undesirable due to the discomfort it causes to the patient, as well as the potential for contribution to thermal or cardiovascular dysregulation. The pathophysiology behind the occurrence of shivering is not well understood, and as a result, the most effective agent for the prevention of shivering in patients receiving spinal anesthesia has yet to be determined. Our scoping review examines 13 studies comparing the efficacy of ketamine versus placebo or other drugs in the management of perioperative shivering.

Our review found that dosages of ketamine ranging from 0.2 mg/kg to 0.5 mg/kg may be effective in the prevention of perioperative shivering [3,6-8]. Another study demonstrated that using a 0.3 mg/kg bolus with 0.1 mg/kg per hour infusion was effective in the management of shivering [4]. However, the remaining studies reviewed demonstrated no difference between agents [2,5,9,14] or another drug to be superior [10-13]. While some studies did show no significant difference between agents or another drug to be superior, this did not mean ketamine was entirely ineffective and still may be considered in shivering management dependent upon patient condition and needs.

There are other factors to be considered when selecting an anesthetic agent. One such consideration is the patient population. One special population of interest is pregnant females because the ideal agent will be safe for both the patient and the fetus. In some studies, ketamine has shown promise as a prophylactic anesthetic agent for the prevention of shivering in females undergoing CDs [6] while not demonstrating any detrimental effects on the neonate [4]. However, there are also data demonstrating that ketamine did significantly decrease the incidence of shivering [2,5] in this population but did have a significantly decreased shivering intensity [2]. A decrease in the intensity of shivering likely decreases metabolic demands and oxygen consumption when compared to shivering of greater intensity. In patients undergoing CD, Azam et al. [7] found ketamine to be more effective than tramadol in the prevention of shivering; however, in other types of surgery, data have varying results for the efficacy of tramadol versus ketamine. More research is needed to determine if ketamine is more effective in the pregnant population alone.

The identification and validation of options for the control of shivering in spinal anesthesia will allow doctors the ability to choose which measure best suits their specific patient. The potential for undesirable side effects needs to be considered. For example, ketamine has been found to induce dose-dependent hallucinations [15,16]. This is a potentially important consideration in patients who are prone to delirium. Other potential side effects of ketamine included post-anesthesia drowsiness [13], but it has also been demonstrated that ketamine is less likely than alternatives to induce nausea, vomiting, respiratory depression, or bradycardia in a 13-study meta-analysis [15]. Sarshivi et al. [2] and Ramanathan et al. [14] found a lower incidence of bradycardia in patients who received ketamine, but the difference was not found to be significant. Hypotension is an especially dangerous adverse effect of some anesthetic agents, but in one study, ketamine had a notable decrease in the incidence of hypotension when compared to ondansetron and meperidine [14].

Because the pathophysiology behind the occurrence of shivering during spinal anesthesia is still poorly understood, there are significant opportunities for further research into both its causes and preventative measures. Of the studies consulted for this paper, many included only small numbers of patients in each experimental group, sometimes as few as 30-60 patients; small groups lead to many of the papers reporting statistically insignificant results, so larger studies could lead to more definitive measures of the efficacy of ketamine in comparison to other measures. Ideally, large randomized controlled trials of a specific type of surgery will be used to help reduce surgery type as possible confounding variables.

Side effects of ketamine and alternative measures are potentially severe, and as such we are also suggesting further research into the optimal dosages and potential drug-drug interactions that may contribute to the occurrence of undesirable side effects such as nausea, vomiting, and hallucinations. However, potential positive benefits beyond shivering should be considered as well. For example, one study found the benefits of ketamine extend beyond the operative period, with it demonstrating a reduction in the opioid requirement within the first 24 hours following completion of a non-elective CD [5]. While research regarding the ability of ketamine to decrease the need for opioid pain management post-operatively is limited by the subjectiveness of pain measurements, agents that are effective for anesthesia, prevention of shivering, and pain control simultaneously are beneficial to research as a means of preventing polypharmacy and improving patient safety.

Timing of medication administration and combinations of medications are also considerations in the management of shivering. This review examines the prevention of ketamine as a single agent in the prevention of shivering; however, Dash et al. [17] compared ketamine, butorphanol, and fentanyl as abortive agents in patients who developed grade 3 shivering that were maintained for at least three minutes. All three agents were shown to be equally effective in controlling shivering by the 10-minute mark, with butorphanol having the quickest onset, but not by a significant margin [17]. However, only the fentanyl group had the recurrence of shivering at a significant rate compared to ketamine and butorphanol [17]. Further research is needed to explore the nuances of the ideal timing of medication administration when used as an abortive agent versus a preventative agent.

Combinations of medications should also be a consideration when choosing the ideal pharmacological regimen. Combinations have the potential benefit of being able to use lower dosages of multiple drugs. This helps reduce the incidence of dose-dependent side effects while possibly increasing desired effects via multiple pharmacologic pathways. One randomized control trial found ketamine plus tramadol to be more effective than tramadol alone in the prevention of shivering [18]. Research into complementary drug combinations may find the use of multiple agents to be ideal if they are dosed to maximize desired effects while minimizing side effects; however, using more drugs increases the risk of drug-drug interactions.

While this review does present ketamine as one of several viable agents in the prevention of shivering, there are several limitations to this review. Studies included in this review evaluated ketamine and shivering in a variety of different surgeries. Different degrees of exposure are present in different procedures that may predispose a patient to shivering. Different studies utilized different dosages and medication timing. There is also a lack of control in that different combinations of other anesthetics and drugs perioperatively are not accounted for in these studies and may be confounding factors. Not all studies reported hemodynamic changes for the evaluation of secondary outcomes. Many of the studies utilized small experimental groups, potentially increasing the risk for type II errors. Finally, as this article is not a meta-analysis, neither bias analysis nor statistical analysis was performed between the included studies. Large, randomized controlled trials consisting of patients undergoing the same procedure with similar drug regimens and dosing would allow for better conclusions to be drawn in regard to the efficacy of ketamine and other agents in the prevention of perioperative shivering. A large meta-analysis would allow for a better comparison of results between individual studies.

## Conclusions

Perioperative shivering is a distressing phenomenon during spinal anesthesia that can lead to significant physiological complications. Ketamine has been shown to be a potentially effective prophylactic measure against this complication. Notably, ketamine has been demonstrated to prevent or reduce the duration of shivering and to promote the maintenance of cardiovascular and respiratory stability. Dosages of 0.2-0.5 mg/kg with or without a subsequent infusion of 0.1 mg/kg per hour may aid in the prevention of perioperative shivering.

However, there are several other pharmacological agents that also may be equally effective. As there is no agent proven to be best, several other factors must be considered when choosing an agent. Patient population, pregnancy status, allergies, hemodynamics, degree of desired sedation, allergies, and provider familiarity with agents all may play a role in determining the best drug to use. Further research is needed to determine ideal dosages, drug timing, and potential synergy of drug combinations.
